# How Do Dieticians on Instagram Teach? The Potential of the Kirkpatrick Model in the Evaluation of the Effectiveness of Nutritional Education in Social Media

**DOI:** 10.3390/nu13062005

**Published:** 2021-06-10

**Authors:** Łucja Zielińska-Tomczak, Piotr Przymuszała, Szymon Tomczak, Izabela Krzyśko-Pieczka, Ryszard Marciniak, Magdalena Cerbin-Koczorowska

**Affiliations:** 1 Department of Medical Education, Poznan University of Medical Sciences, 7 Rokietnicka St., 60-806 Poznan, Poland; zielinskatomczak@ump.edu.pl (Ł.Z.-T.); pprzymuszala@ump.edu.pl (P.P.); rmarcin@ump.edu.pl (R.M.); mcerbin@ump.edu.pl (M.C.-K.); 2 Department of Pharmaceutical Chemistry, Poznan University of Medical Sciences, 6 Grunwaldzka St., 60-780 Poznan, Poland; 3 Department of Pediatric Diabetes and Obesity, Poznan University of Medical Sciences, 27/33 Szpitalna St., 60-572 Poznan, Poland; ikrzysko@ump.edu.pl

**Keywords:** nutritional education, health promotion, Kirkpatrick Model, dieticians, Instagram

## Abstract

The growing popularity of health education on social media indicates the need for its appropriate evaluation. This paper aims to present the potential of the Kirkpatrick Model (KM) with New World Kirkpatrick Model (NWKM) additions to evaluate the nutritional education provided by dieticians via Instagram. Instagram profiles of ten dieticians providing nutritional education for their followers were analyzed in March and April 2021. The study sample included profiles of both macro- and micro-influencers. The analyzed quantitative data included Instagram Engagement Rate and the number of likes and comments per post. The qualitative analysis of the comments was performed following the theoretical framework provided by the KM and NWKM. Collected data showed followers’ satisfaction, commitment, and relevance of the presented content, fulfilling the Level 1 of NWKM. Level 2 of NWKM was represented by 4 out of 5 dimensions (knowledge, attitude, confidence, commitment). No comments were found only for skills. Both Levels 3 (Behavior) and 4 (Results) of the KM were met. However, the use of the NWKM for them seems limited. The KM can be used to evaluate nutritional education on social media. The NWKM additions seem applicable mostly for Levels 1 and 2.

## 1. Introduction

In the last decade, we can observe an increased interest in the Internet as a source of information with social media (SM), becoming a ubiquitous factor in everyday life of humans [1,2]. According to the SM definition by the Merriam-Webster dictionary [3], these interactive technologies are orientated toward creating and sharing content among members of Internet communities. SM meet many needs, including the need for belonging, acceptance, and social interactions [4], which is why they have nowadays become an integral part of daily Internet usage, with over 3.6 billion users worldwide in January 2020. This value will probably reach even up to 4.41 billion in 2025 [5]. Health educators and promoters also have recognized SM’s potential to deliver easily accessible and enjoyable content for the recipients. Social media in health promotion are gaining popularity due to their availability, regardless of the existing physical barriers preventing direct health education. The attractiveness of running pro-health campaigns via social media is also associated with lower costs of conducted actions [1,6,7].

Nutritional education has also found its place on Instagram. As Kawiak-Jawor et al. [8] reported, there is a belief among social media users that it is food that has the most significant impact on health. This offers the potential for dietitians to create educational content. According to the National Center of Nutritional Education affiliated with the Polish Ministry of Health, which aims to promote a healthy lifestyle, education in the field of changing eating habits is a more effective lifestyle-changing tool than, for example, preparing meals according to a composed diet [9]. Previously conducted studies proved the potential of SM in nutrition education [10]. However, as Nour et al. [10] indicated, many of those solutions were either evaluated with nonvalidated tools or based solely on short-term outcomes measurements.

Similarly, despite the observed enormous potential of social media in health promotion, there is a lack of appropriate tools to assess their effectiveness in improving public health outcomes [1]. As the definition of health education indicates, this process is not only about disseminating information but about creating learning opportunities through “fostering the motivation, skills, and confidence (self-efficacy) necessary to take action to improve health” [11]. Therefore, evaluating the effectiveness of health education should not be limited only to assessing the change in participant knowledge. We should also seek tools that allow verifying other elements such as motivation and self-efficacy.

The potential for their evaluation can be sought in the Kirkpatrick Model (KM) and its additions provided by the New World Kirkpatrick Model (NWKM) [12]. There are several reports on the use of KM in assessing the effectiveness of health education, for instance, in the form of workshops and computer games [13,14,15,16,17]. For example, Grant et al. [13] used KM to evaluate the effects of glycemic index education on lowering dietary glycemic index among people living with type 2 diabetes. They used the first three levels of KM (satisfaction, knowledge increase, and behavior change), which they assessed using a questionnaire of their authorship (Glycemic Index Questionnaire). In their study, KM has proven to be an effective evaluating tool. The research of diabetes medication-related workshops, which pharmacists performed, also indicates the possibility of using KM in health education [17]. The use of KM in the evaluation of computer games in asthma education showed the effectiveness of this educational method at all four assessment levels [14].

However, to our knowledge, no evaluation of health education conducted using social media has been conducted so far. Therefore, this study aims to present the potential of KM with added NWKM dimensions to assess the effectiveness of health education on nutrition provided by dieticians via social media on the example of Instagram. 

## 2. Materials and Methods

Due to the strong emphasis on trying to change eating behavior as early as possible in life [18], this study focused on Instagram, which users are most commonly the younger generation members (18–34 years) [19]. 

The study was conducted in February and April 2021 on the Instagram social network. During the study, a sample of ten Instagram accounts led by dieticians and containing educational content was chosen and analyzed. Profiles were searched using the hashtag #dietetyk (#dietician in Polish). Then the profile description was analyzed (“BIO”) whether the influencer declares having a degree in dietetics. The accounts varied in the number of followers, and both the macro and micro-influencers were included in the sample. The profile analysis was carried out on 6–12 April 2021. The data was anonymized, subsequent profiles were encoded and assigned in ascending order (e.g.1st Instagram profile was assigned as IG1, etc.).

### 2.1. Theoretical Framework

The Kirkpatrick Model (KM) is a well-established model of training evaluation based on four levels:

- Level 1 (L1): Reaction, which assesses the extent to which participants find the learning experience favorable,
- Level 2 (L2): Learning, which describes changes in participants knowledge, skills, and attitudes),
- Level 3 (L3): Behavior, which indicates whether participants change their behavior and apply what they have learned,
- Level 4 (L4): Results, which measure the occurrence of intended outcomes [12].

This model has been developed to evaluate training and shift the focus in evolution to the results achieved [20,21] and has inspired the development of several other evaluation models [20,22,23]. Hence, it is frequently used as a popular evaluation method [20,24,25], and it is often cited in research papers [26].

Nonetheless, some of its drawbacks should also be acknowledged. For instance, Holton [22] and Alliger and Janak [27] indicated the model’s constraints concerning the model hierarchy. Moreover, there are no empirical studies to confirm the essence of the hierarchical character [20]. Kirkpatrick and Kirkpatrick [28], presenting the updated model of evaluation (New World Kirkpatrick Model), indicate that there is no need to evaluate this aspect. Despite the limitations, KM is widely used and considered helpful in assessing the training process [26]. The KM tool, like any model, has strengths and weaknesses, but researchers highlight that KM is more suitable than other models [29].

**Figure 1:**
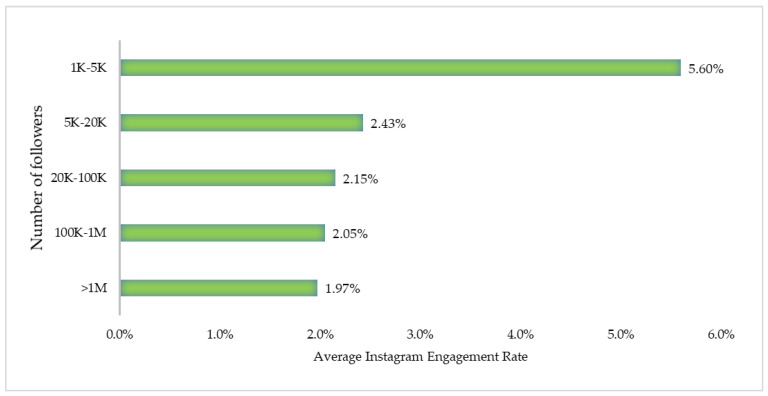
Average engagement rate on Instagram.

The Kirkpatrick Model (KM) may provide the potential for assessing the effectiveness of educational activities in social media. In this study, the Kirkpatrick Model was used, where applicable, together with the new elements and clarifications provided by the New World Kirkpatrick Model (NWKM), which allows for an earlier assessment of educational effects. In the original model, in order to be able to assess the outcomes, one would have to wait for them, which seems difficult to obtain in the case of social media. On the other hand, the NWKM provides tools that, among others, can be used to assess whether the learner is on the right track to achieve the intended outcomes. In the NWKM, Level 1, which previously focused only on participants’ satisfaction, was supplemented with their engagement (active involvement) in the content and its relevance to them, indicating whether the content will be useful or applicable to the participants. Similarly, the dimensions within the original KM, namely knowledge (summarized as “I know it”), skills (“I can do it right now”), and attitude (“I believe it will be worthwhile”), were completed with confidence (“I think I can do it”) and commitment (“I will do it”). Additions on the Level 3 included critical behaviors, required drivers, and on-the-job learning. Given that the decision which behaviors are critical (having the biggest impact on the outcomes) should belong to the trainers (as experts) and not the learners, we decided not to go into details with them in this research. Moreover, since we were not able to reliably identify them from followers’ comments, it would also be difficult to indicate required drivers that by definition “reinforce, monitor, encourage and reward performance of critical behaviors” as well as elements of the on-the-job learning. Consequently, in view of the aims and methodology of the study assuming analysis of followers’ comments, we evaluated Level 3 with a bigger focus on their self-observed behavioral changes and implementation of the dieticians’ recommendations. Finally, the addition to the Level 4 introduced leading indicators as “short-term observations and measurements that suggest that critical behaviors are on track to create a positive impact on the desired results.” Again, due to the impossibility to reliably identify the critical behaviors, we put more focus on the intended outcomes of followers, which were attributed by them in the comments to the provided educational content. However, given that follower’s comments are mostly a response to previous recommendations of a dietician, we believe that including short-term observations on them also seems justified. Figure 1 presents the division of dimensions of KM.

### 2.2. Qualitative Analysis

The qualitative analysis of collected data was conducted by two analysts (ŁZT and PP). They independently evaluated the comments left on educational posts and then assigned them to the matching assessment levels according to the KM and, where applicable, also NWKM (L1–L4). Other types of comments that did not correspond to any of the levels were not taken into account during further analysis. An authorial Microsoft Excel form was used for the analysis. Its draft is presented as Table 1, along with exemplary comments assigned to the corresponding NWKM levels.

The agreement level between the researchers was measured with kappa index using the GraphPad website [30]. It showed their high agreement with kappa = 0.833 (95% confidence interval (CI) 0.739–0.926) [31]. Any differences that occurred in assigning comments to the KM levels were further discussed between them until the consensus was reached. 

### 2.3. Quantitative Analysis

For the evaluation of the NWKM Level 1, we also used the quantitative analysis through the Instagram Engagement Rate (IER). IER is a factor that indicates the number of responses and interactions generated by a given content in social media. The calculation method of engagement rate is characteristic and specific for each of them [32]. In this study, we used the Phlanx: Social Media Marketing Platform to evaluate the Instagram Engagement Rate [33]. Additionally, the numbers of likes and comments under posts were analyzed.

## 3. Results

Among ten Instagram profiles run by dieticians that were included in the analysis, five had a range of 1000–100,000 followers (micro-influencers), and five had over 100,000 followers (macro-influencers) [34]. Nine profiles were run by women and one by a man. All profiles were led by dietetics graduates, and two of them additionally held a doctoral degree.

### 3.1. Kirkpatrick Model L1

#### 3.1.1. Satisfaction

Followers leaving likes under posts were showing their satisfaction with the content provided. The average number of likes in the analyzed period varied between 269 and 4292 likes per post. Followers’ satisfaction with the educational activities was also visible in the number of comments under the posts, which ranged, on average, between 32 and 110 per post (Table 2).

Through the comments, followers were often thanking for providing interesting and knowledgeable educational content. They praised the concise method of transferring knowledge, which is extremely important when educating in this type of social media. 

IG3: “As always, a great post. The knowledge given briefly and succinctly. You have big talent. And, of course, knowledge. 😃 Thank you!”

IG1: “WOW 🔥🔥🔥 this is one of the best posts I have read on insta [slang abbreviation for Instagram]”

IG3: “As always, a lot of knowledge in the post, mega! 😍”

IG1: “Wow, great post, great form! I haven’t seen anything like that on Instagram, it definitely brings freshness, and for me personally, the information presented in this way is more memorable. Bravo!”

#### 3.1.2. Engagement

IER is a reliable measurement of followers’ engagement (Table 2). The average IER on the profiles varied between 1.63% and 3.89% of followers. The exception is the IG3 profile, where this value reaches 15.19%.

Participants following the profiles of educating dieticians also present their commitment and active involvement by asking additional or clarifying questions, among others.

IG1: “And what does the physical activity change? Do we burn fat faster instead of muscles? […]”

IG1: “Do we count the amount of protein according to the total body weight or lean weight?”

#### 3.1.3. Relevance

On the basis of comments, it is also possible to assess whether the information provided is perceived by followers as important, meeting their needs and whether it will be useful or applicable for them. 

IG4: “thanks a lot, I save it [the post] I will know what to put on the plate during #healthchallenge I always had a problem with which #vegetables are in the season!”

IG3: “Great post. A lot of people around me accuse my friend of having Insulin Resistance because she eats too many sweets. Now, thanks to your knowledge, I will support her even better!”

IG5: “A very clear message, this is what I was looking for and which dispels all my doubts … time for research.”

IG2: “A much-needed topic! I am trying to fight on this topic now”

### 3.2. Kirkpatrick Model L2

#### 3.2.1. Knowledge

Participants of the community gathered around dieticians indicated in the comments that they learned a lot from the content and presented educational activities increased their level of knowledge. They also indicated that visual representations of individual content help to consolidate knowledge, which they already had.

IG2: “I love your profile! I learned a lot from it”

IG3: “I always learn new valuable information from your posts” 

IG1: “In a way, I guess I knew, but to see is different. Thanks for the experience”

The presented content teaches followers the rules of rational eating and a properly balanced diet. They are also a source of knowledge about seasonal food. 

IG5: “Super explained 🙂 I finally know how many kcal I need to lose weight”

One of the more popular topics that dieticians educate about is insulin resistance. It turns out that the followers perceive this topic as very important, but simultaneously it is often misunderstood. The content provided by the dieticians allowed to improve the level of knowledge of the recipients in this field. 

IG3: “It’s great that you help to organize the knowledge about Insulin Resistance so nicely 🙌”

IG3: “I was surprised by almost everything, I am not familiar with insulin resistance because I did not have much contact with it, but I heard a lot of myths! Now I’ll be smarter” 

Dieticians on their profiles discuss the existing myths and misconceptions about nutrition and indicate what is true and what is false. As it turns out, they are of great help to followers who often believe in commonly reproduced myths.

IG5: “Until now, I believed in butter and coconut oil”

#### 3.2.2. Skills

No comments were found that would indicate the impact of Instagram profiles on followers’ skills. This may be caused by the nature of the interaction between the Instagram users and the way the content is shared between them. As a result, analyzed educational activities carried out by the dieticians with the use of Instagram did not focus on skills but on providing information, knowledge, and changing the health habits of the followers. 

Additionally, the lack of identified activities focused on skills can result from the limited number of analyzed profiles, which was discussed further in the Limitations section. However, if this content appeared, especially in other social media, the KM with NWKM additions would still allow its evaluation. 

#### 3.2.3. Attitude

Comments left by the followers participating in the health education indicate that they accept the solutions proposed by the dieticians and believe that their implementation will be beneficial and worthwhile. 

IG3: “I definitely must try it” 

IG4: “this second set sounds pretty interesting, and I will definitely try it out”

#### 3.2.4. Confidence

Due to the Instagram activity of the dieticians, followers also begin to believe they will be able to implement what they have learned. 

IG2: “Maybe I will finally succeed” 

IG5: “A very clear message, this is what I was looking for and which dispels all my doubts … it’s time for tests.”

#### 3.2.5. Commitment

Finally, followers indicate real actions that they intend to implement after Instagram health education. They indicate how motivated they are to perform a certain activity or how they intend to do something. The use of phrases such as “I’m rushing”, “I’m doing it tomorrow” indicate that the participants want to implement the change immediately.

IG2: “I’m rushing! I have a terrible problem with sweets 😢”

IG3: “I’m doing it tomorrow”

### 3.3. Kirkpatrick Model L3

Under Level 3, the behavior change of followers is investigated, which again can be traced on the basis of their comments. Followers declare that they apply what they learned from the profiles or attempt to change their lifestyle. 

IG1: “I slowly introduce this habit at home” 

IG2: “This [the profile] really helps me with rational nutrition after bariatric surgery”

Another example is a comment under the post encouraging to freeze the remaining food after Easter and plan light meals for the next days. 

IG2: “Frozen and [light] lunches are planned” 

They also indicate a change in attitudes towards losing weight, including changing not only their eating habits but also those related to physical activity. In this context, followers’ comments may also serve as sources of information on which behaviors they perceive as critical. However, given that identifying those few actions with the biggest potential impact on the outcomes should rather remain the domain of trainers, and not the learners, we did not attempt to analyze it in great detail. 

IG3: “As usual, in point. I also sometimes “diet” like the patients mentioned above. Now I’m smarter. For several weeks I have been changing my habits, not only eating habits. I move regularly, but knowingly, I do not sit at night, as was often the case, I eat wisely, but I’m not obsessively counting calories.”

Similarly, although the chances to analyze followers’ posts in terms of their required drivers remain limited due to the lack of defined critical behaviors and a low number of their comments on what actually motivates and encourages them to change their habits, it seems that the observed Instagram posts could also serve as such sources of reinforcement, support, encouragement, and motivation. An additional reward or motivating factor for followers also becomes the opportunity to show off their successes in the comments.

IG2: “I am planning to limit meat, especially pork-I do not eat beef-in favor of fish, although I must admit that dishes made of chickpeas or other legumes are also delicious if well done. I haven’t eaten a bean burger yet, but I’ll make such a wonder someday” 

IG6: “You’re doing a good job because I haven’t bought a single gram of sweets. The only exception is the poppy seed cake, and it is enough for me to have this ‘atmosphere’.”

IG9: “I’ve been here for 2 months, and I have to thank you for the new lifestyle. Healthy, delicious, and colorful and what’s important to me: you convinced me to a few dishes that I never ate normally”

### 3.4. Kirkpatrick Model L4

In the comments under the posts, we can see how they contribute to the occurrence of targeted outcomes or trace how followers’ behaviors are on track to achieve the desired health outcomes. Due to the application of the NWKM approach, we can assess the effectiveness of Instagram educational activities on this basis. 

IG2: “It took me a while to understand it, but today I agree with it at 1000% ❤ losing weight is not a race, slowly, healthy and to the goal”

IG3: “On Sunday, I ate a piece of my daughter’s birthday cake, and I’m still losing weight. I feel very good with it”

## 4. Discussion

The dynamic of the popularity of health education is gaining momentum [35]. There is a growing number of profiles led inter alia by dieticians or health lifestylers, aimed at educating the public opinion. The content raised by them concerns diets, nutrients, and education in the field of healthy eating [36]. 

The presented study showed the potential of using KM and NWKM as a tool for the evaluation of health education conducted with the use of the Instagram social network. As mentioned above, its effectiveness has already been proven in the evaluation of workshops and computer games in the field of health promotion [13,14,15,16,17]. However, to the best knowledge of authors, this is the first study in the context of social media, so the opportunities for data comparison are limited. 

To assess the effectiveness of these activities, a properly adapted tool for evaluating this type of education is needed. Evaluating all four levels of KM gives a whole picture of the effectiveness of the conducted educational activities, starting from participants’ satisfaction with the training, through the analysis of knowledge or behavioral changes, to the real effects of the education [12]. The effectiveness evaluation allows educators to improve their content, adapt it to the needs of recipients, or monitor the progress in achieving the intended outcomes of health education. The imperfections detected in time allow to make modifications to the conducted educational activities [1]. Participants of the educational interventions conducted by dieticians express satisfaction with the training, show commitment, and comment on its relevance for them (L1). They inform about the increase in knowledge of the topics covered on the profiles and changes in their attitudes, confidence, and commitment towards the intention to use the acquired knowledge in practice or change their habits L2). They also discuss changes in behavior that occurred as a result of the impact of the presented content (L3). Finally, also L4 can be assessed via signals suggesting that the behaviors of participants of educational activities are on the way to achieve the intended outcomes.

In the updated approach to KM [28], the essential character of the L4 evaluation is emphasized. Focusing on the L4 during the design of educational intervention provides opportunities to achieve this level as efforts are focused on it and, as a result, the expected outcomes of the intervention are defined at an early stage. In guides or blogs dedicated to the effectiveness of holding accounts on Instagram, it can be read that the key to success in social media is the correct definition of the purpose of the activities carried out [37,38]. When L4 is difficult to measure, an attempt should be made to evaluate L3 as it has been shown that L3 allows to directly predict L4. Moreover, the feedback at the L3 level paints a picture not only about the change in the behavior of the participant but also gives information on how the levels L1 and L2 were realized [39].

The evaluation of these levels is possible through the use of both quantitative and qualitative methods. Similarly, according to Kampka [40], Instagram content can also be analyzed using quantitative and qualitative methods. Quantitative indicators can be helpful in analyzing L1, like, for example, an assessment of the outreach of presented contents in the field of health education [41]. Also, the popularity of the channel, the measurement of followers’ activity, or the involvement in the presented content may be assessed [1]. The latter seems to be a very important parameter, as it is the factor that is most strongly related to the impact of influencers on a given community [42]. However, to perform a complete analysis (L1–L4), a qualitative study of the comments should also be performed. They constitute a quasi-source of feedback on the activities carried out. In the study of Grant et al. [13], the qualitative methodology was also used as an element of health education evaluation developed within KM. 

Bates [20] points out that the four-stage model presents a simplified view on the effectiveness of conducted educational activities. Among the elements that are not subject to evaluation are, among others, learning culture, goals, values of learners, or the adequacy of material resources itself, which can influence the outcomes of the training. In education conducted with the use of Instagram, it is worth noting that, by definition, there is a consistency of the values or goals of influences and their followers. The archetype that fits influencers corresponds to their beliefs and values and at the same time directs their activities to a specific group of recipients whose goals will be similar [43]. It is worth extending health education research through Instagram on the study of archetypes of health educators. 

## 5. Limitations

The study has several limitations. Firstly, the first author’s bias cannot be ruled out due to her own opinions on the subject. However, to reduce the subjectivity of the data analysis, the researcher’s triangulation was used, which allowed decreasing this risk. Then, only ten accounts led by dieticians from Poland were analyzed, which does not allow drawing broader conclusions for the entire population. However, this preliminary study intended to illustrate and indicate the possibilities of using KM in nutritional education in social media. Repeating the analysis for accounts maintained by influencers from other countries will allow for the provision of further data that will allow extrapolation of the results. Then, it cannot also be excluded that the data obtained from the comments are based on followers’ self-reporting and were not verified in any other way. Although the followers did not actually have any reason to write in the comments something that would not be true, exploring their perspective allows not only better getting to know their motivation to use Instagram profiles focused on health education, but also factors that affect the improvement of motivation, skills or self-efficacy in taking actions related to health.

Finally, to the best of our knowledge, consistency analysis of KM has not been performed yet. However, KM is frequently used as a popular evaluation method, often cited in research papers. It may mean that this method has been tested well. Consideration should be given to consistency analysis of KM in future research.

## 6. Conclusions

The provided example of the Instagram social network allows concluding that the Kirkpatrick Model with the New World additions carries the potential for the evaluation of health and nutritional education conducted with the use of social media. However, the NWKM application for Levels 3 and 4 seems to be limited without the additional involvement of influencers in defining critical behaviors. The ability to assess all four levels of KM can give a picture of the extent to which this type of health promotion is achieving its goals. Also, a preliminary evaluation of the educational activities of dietitians on their Instagram accounts indicates that this type of education can have a big impact on their followers. Our study shows that nutritional education on Instagram is perceived by followers as satisfying, engaging, and relevant (Level 1), as well as may contribute to improving their knowledge, attitude, confidence, and commitment to changing lifestyle (Level 2) and translate into pro-health behaviour changes (Level 3). The results also show that there are short-term indicators that followers are on a good way to achieve the desired results (Level 4). Although due to the lack of identified posts teaching skills, we found no comments on the impact of educational content on followers’ skills, if a skill-focused content appeared on social media, the KM with NWKM additions would still allow its evaluation.

Due to the growing popularity of activities in the field of dietetics and health education, indicating that KM may be used to evaluate these activities allows adopting its use by Influencers conducting these activities. Proper assessment of education may translate into improving its quality.

Further research should attempt to extend this study’s results by contacting both Influencers and Followers and collecting data from them, for instance during individual in-depth interviews. It will allow learning about their perspectives and attitudes towards nutritional education and a complete assessment (especially in terms of 3 and 4 KM levels) of educational activities conducted by them.

## Figures and Tables

**Table 1:** The comment evaluation form used during the study with examples.

NWKM Level.	Dimensions (Where Applicable)	IG Code	Comments
Level 1: Reaction	Customer Satisfaction	IG5	“Finally, this is super explained! Brilliant post”
	Engagement	IG1	“Do we count the amount of protein according to the total body weight or lean weight?”
	Relevance	IG4	“And that’s what I understand. I was just looking for such inspiration. Finally a profile that meets my expectations”
Level 2: Learning	Knowledge	IG5	“Great, thanks! 🤗 I finally know how many kcal I should eat per day”
	Skill	-	-
	Attitude	IG9	“The super cocktail 😍 I will definitely make 😋”
	Confidence	IG2	“Maybe I will finally succeed”
	Commitment	IG10	“I’m going to do a test in May!”
Level 3: Behavior		IG8	“Thanks to you, I started to get up earlier. In the beginning, it was difficult, but now I get up at 5–6 without any problems, and go to bed at 21–22. The difference is amazing”
Level 4: Results		IG10	“We have been following the recommendations on the right since the beginning of the year. 🙌 The effects exceeded our expectations.”

IG—Instagram; NWKM- New World Kirkpatrick Model.

**Table 2:** Characteristic of Instagramers’ profiles.

IG ^1^ Code	Followers Number	Range	IER ^2^	Likes ^3^	Comments ^3^	Engagement ^4^
**IG1**	36,165	micro	3.15	1149	37	higher
**IG2**	225,376	macro	1.87	4292	81	lower
**IG3**	2031	micro	15.19	269	59	higher
**IG4**	65,795	micro	2.48	1673	43	higher
**IG5**	65,227	micro	3.89	2609	48	higher
**IG6**	135,217	macro	1.63	2165	78	lower
**IG7**	24,296	micro	2.33	649	32	higher
**IG8**	118,837	macro	3.23	3996	110	higher
**IG9**	231,997	macro	1.72	4153	110	lower
**IG10**	51,367	macro	2.11	1124	28	lower

^1^ IG—Instagram. ^2^ IER—Instagram Engagement Rate. ^3^ per post. ^4^ profile’s engagement relative to the average.

## Data Availability

Data are contained within the article.
